# Cancer Disparities and Black American Representation in Clinical Trials Leading to the Approval of Oral Chemotherapy Drugs in the United States Between 2009 and 2019

**DOI:** 10.1200/OP.20.01108

**Published:** 2021-05-11

**Authors:** Veronica B. Ajewole, Oyinkansola Akindele, Uzoamaka Abajue, Okwuoma Ndulue, Jazzmin J. Marshall, Yhenew T. Mossi

**Affiliations:** ^1^ Department of Pharmacy Practice and Clinical Health Sciences, College of Pharmacy and Health Sciences, Texas Southern University, Houston, TX; ^2^ Department of Pharmacy, Houston Methodist Hospital, Houston, TX; ^3^ Houston Methodist Academic Institute/Weill Cornell Medical College, Houston, TX

## Abstract

Over the past 10 years, oral chemotherapy made up about half (45.6%) of all US Food and Drug Administration (FDA)–approved oncolytic and hematologic medications. Given the disparity in incidence and mortality rate because of certain cancers among Black Americans (BAs) in the United States, a review of BA’s representation in the clinical trials that lead to the development and FDA approval of oral chemotherapy drugs becomes imperative. The objective of this study was to evaluate the reporting of race and inclusion of BA in clinical trials that led to the approval of oral chemotherapy medications by the FDA from 2009 to 2019 in the United States. Additionally, we evaluated the inclusion of BAs in clinical trials of three cancer types with the highest disparity rates among BAs (lung, breast, and prostate).

**METHODS:**

A retrospective review of all FDA-approved oral chemotherapy drug from 2009-2019 was obtained using the FDA’s Hematology/Oncology Approvals & Safety Notifications website. Reports of racial and demographics inclusion were obtained from the clinical trials registry.

**RESULTS:**

Primary outcome: 142 clinical trials led to FDA approval of 81 oral chemotherapy agents between 2009 and 2019, among which 74 (52%) reported on at least one race and were included in our analysis. 35,933 participants were enrolled in these 74 clinical trials, among which 25,684 (71.47%), 6,061 (16.87%), 889 (2.47%), and 826 (2.30%) were White, Asian, Black, and Hispanic, respectively. BAs were also under-represented in the clinical trials of three cancer types with the highest disparity rates among this population.

**CONCLUSION:**

BAs were under-represented in clinical trials leading to FDA approval of oral chemotherapy drugs. There should be more BAs in cancer clinical trials to increase the generalizability of the results, improve outcomes, and eventually close the health disparity gap among this patient population.

## INTRODUCTION

Oral chemotherapy agents have become more prevalent in treating various cancers. Between March 2009 and December 2019, the US Food and Drug Administration (FDA) approved 81 oral chemotherapy agents with some oral chemotherapy agents having multiple indicatons.^1^ The increased occurrence of oral chemotherapy usage has been attributed to ease of administration and improved quality of life.^2^ Although many oral chemotherapy agents have been approved by the FDA, patient populations from diverse racial-ethnic background inclusive of the US population are often not adequately represented or reported in clinical trials,^3^ thus leading to racial disparity among oncology patient representation in clinical trials.^3^ According to Healthy People 2020, health disparity is a health difference that is intricately linked with social, economic, and/or environmental disadvantage.^4^ Racial disparities in health care can have a significant impact on patient outcomes and survival.^5^ According to the National Institutes of Health (NIH), health disparity populations include Black or African American, Hispanic or Latino, American Indian or Alaska Native, Asian American, Native Hawaiian, and other Pacific Islander.^6^ Ethnic minorities, particularly Black Americans (BAs), have higher incidence for many cancers such as lung, prostate, and colon, which occur in 62.4, 173.0, and 45.7 BAs per 100,000 people, compared with 63.5, 97.1, and 38.6 White per 100,000 people, respectively.^5,7^ Unfortunately, BAs also have higher mortality rates in cancers such as prostate, where mortality rates are more than double any other racial group.^7^ More specifically, among BAs, mortality rates for lung, prostate, and colon cancers are 43.5, 38.7, and 19 per 100,000 people, respectively, whereas mortality rates among White are 43.4, 18, and 13.8, respectively.^7^ Although the incidence of breast cancer is lower in BAs compared with White (126.7 *v* 130.8 per 100,000 people), breast cancer causes significantly higher mortality in BAs compared with White (28.4 *v* 20.3 per 100,000 people).^7^ Lung and colon cancers are the second and third most common cancers after prostate cancer in men and breast cancer in women.^7^ Many clinical trials focus on representing the demographics of the United States without considering the increased cancer burden in BAs.^8^ Although cancer rates are higher among BAs, BAs in clinical trials remain low.^3^ This poses a challenge because genetic variation among ethnic minority groups can be a determinant of health outcomes.^3^

In 1993, the NIH Revitalization Act was passed by US congress to lead the federal agency’s inclusion of women and ethnic minorities in clinical studies to promote clinical research equity.^9^ As a result, over the past 27 years, gender participation has leveled off with 72% female participation in clinical trials in 2019.^10^ However, clinical trials continue to lack racial and ethnic diversity. As of 2019, BAs accounted for 13.4% of the total US population and only 9% participated in clinical trials, whereas White represented 76.5% of the US population and 72% participated in clinical trials.^10,11^

Understanding the safety and efficacy of drugs for all patients hinges on the participation of diverse racial and ethnic subgroups. When drugs are tested in clinical trials, it is believed that the population that the drug was tested in mirrors the patient population who will ultimately receive treatment with the drug. When there is skewing in the clinical trial population relative to the population of patients that actually receive the drug, extrapolation of clinical outcomes across all patient population becomes very challenging. This disproportionate participation of BAs in clinical research limits the ability of BAs to fully benefit from biomedical research advances (including access to cutting-edge therapies) and contributes to racial health disparities.^12-14^ Clinical trials with diverse populations are needed to better predict the generalizability of clinical outcomes among minority population.^5^ Although the NIH has placed regulations that require minority representation in clinical trials, enrollment in clinical trials remains disproportional to prevalence of disease among BAs.^3,5^

Awareness of the current trend of cancer disparities and BA representation in clinical trials leading to the approval of oral chemotherapy drugs in the United States becomes imperative. This will inform stakeholders, policymakers, health officials, and the general public in informed decision making that will enhance BA participation and representation in oral chemotherapy clinical trials. This will be a step in the right direction to closing the health disparity gap in cancer care for BAs. Thus, in this paper, we present our review of clinical trial enrollment demographics for FDA-approved oral chemotherapy medications between 2009 and 2019.

## METHODS

### Study Cohort

We retrospectively reviewed all oral chemotherapy drugs approved by the FDA from March 2009 to December 2019 using the FDA’s Hematology/Oncology (Cancer) Approvals & Safety Notifications website.^1^ We identified the four major races based on US census categories.^11^ Race reporting was obtained from the NIH clinical trials registry,^15^ and also reconciled with the published primary literature for each drug. The data from the NIH clinical trials registry^15^ and published primary literature were also reviewed to identify if the clinical trials enrolled patients predominantly in the United States, International counties including the US, or Non-US–based countries. Based on available literature, we identified trials where there is a known racial subset that would be over-represented because of disease prevalence among certain racial-ethnic population. Additionally, information about industry-sponsored versus cooperative group trials was collected. For drug approvals with more than one clinical trial, we included all trials in our analysis. Data on the US cancer incidence and mortality were obtained from National Cancer Institute's SEER database.^16^

### Statistical Analysis

Of the clinical trials obtained, trials that did not report on at least one race were filtered out. Of the remaining trials, the total number of participants enrolled in each clinical trial was used to calculate the proportion of White, Asian, Black, and Hispanic enrolled in each clinical trial, which is the primary outcome of our study.

The clinical trials data were further analyzed by cancer type with highest mortality in BA population—Lung, Breast, and Prostate Cancer. Although colorectal cancer has a high mortality rate among BA population, the three clinical trials on colorectal cancer did not report racial demographics and thus were excluded from our analysis. Based on these data, the total number of participants enrolled in each clinical trial was used to calculate the proportion of White, Asian, Black, and Hispanic enrolled in each clinical trial. This composite analysis is the secondary outcome for this study. Additionally, we report on clinical trials patients' enrollment locations as predominantly United States, International counties including the United States, or Non-US–based countries as well as racial subset enrollment because of disease prevalence among certain racial-ethnic population and industry-sponsored versus cooperative group trials.

## RESULTS

### Primary Outcome

**FIG 1.**
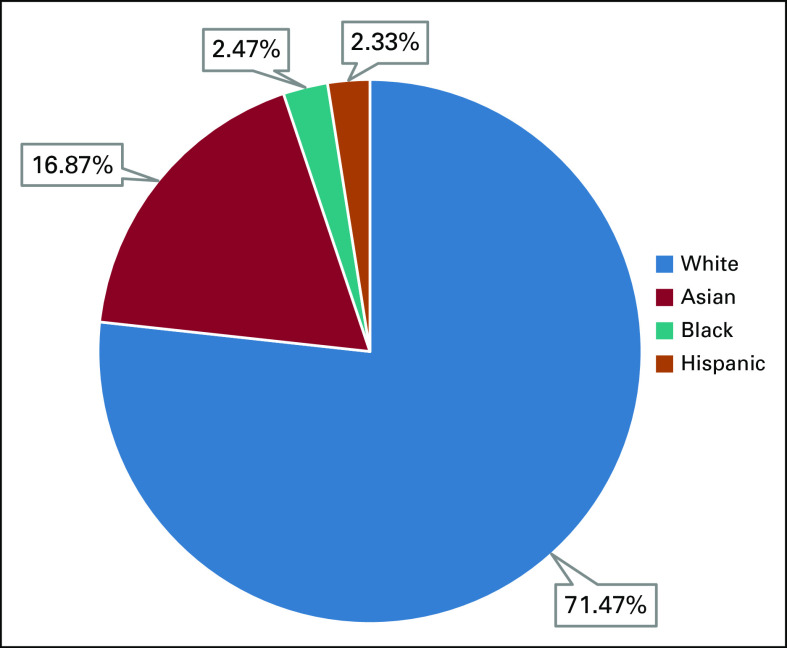
Black American representation in oral chemotherapy clinical trials, 2009-2019.

One hundred forty-two clinical trials led to FDA approval of 81 oral chemotherapy drugs in the United States between 2009 and 2019. Among these 142 clinical trials, 74 (52%) of these reported on at least one race and were included in our analysis. A total of 35,933 participants were enrolled in these 74 clinical trials. Among the 35,933 participants, 25,684 (71.47%), 6,061 (16.87%), 889 (2.47%), and 826 (2.30%) were White, Asian, Black, and Hispanic, respectively. See Figure 1 for details.

### Secondary Outcomes

#### Non–small-cell lung cancer.

**FIG 2.**
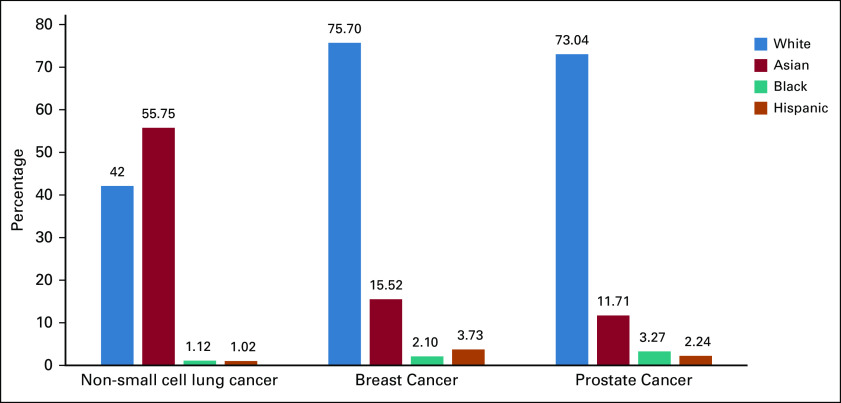
Black American inclusion in oral chemotherapy clinical trials involving top three disparity-related cancers among Black American populations 2009-2019.

Between 2009 and 2019, 21 clinical trials led to FDA approval of 12 oral chemotherapy agents for the treatment of non–small-cell lung cancer. Among these 21 clinical trials, 11 (52%) reported on at least one race and were included in our analysis. These 11 clinical trials had a total of 3,811 participants enrolled with 1,605 (42.11%), 2,125 (55.75%), 43 (1.12%), and 39 (1.02%) being White, Asian, Black and Hispanic, respectively. See Figure 2 for details.

#### Breast cancer.

Between 2009 and 2019, 12 clinical trials led to FDA approval of 11 oral chemotherapy agents for the treatment of breast cancer. Among these 12 clinical trials, eight (66.67%) reported on at least one race and were included in our analysis These eight clinical trials enrolled 7,318 participants with 5,540 (75.70%), 1,136 (15.52%), 154 (2.10%), and 273 (3.73%) being White, Asian, Black, and Hispanic, respectively. See Figure 2 for details.

#### Prostate cancer.

Between 2009 and 2019, eight clinical trials led to FDA approval of six oral chemotherapy agents for the treatment of prostate cancer. Among these eight clinical trials, six (75%) reported on at least one race and were included in our analysis These six trials enrolled 6,876 participants with 5,022 (73.04%), 805 (11.71%), 225 (3.27%), and 154 (2.24%) being White, Asian, Black, and Hispanic, respectively. See Figure 2 for details.

### Other Outcomes

#### Clinical trials patients' enrollment location.

Among all 142 clinical trials, 26 (18%) clinical trials enrolled patients predominantly in the United States. Of these 26 clinical trials, 14 (54%) reported on at least one race. Of these 14 clinical trials, the enrollment rate of White, Asian, Black, and Hispanic ranged from 38.4%-94.8%, 1.8%-30%, 0.52%-7.1%, and 2.2%-8.9%, respectively. Among all 142 clinical trials, 106 (75%) enrolled patients across international counties including the United States. Of these 106 clinical trials, 56 (52.8%) reported on at least one race. Of these 56 clinical trials, the enrollment rate of White, Asian, Black, and Hispanic ranged from 31.9%-98.7%, 0.6%-65.4%, 0.18%-3.2%, and 0.93%-17.2%, respectively. Among all 142 clinical trials, 10 (7%) enrolled patients in Non-US–based countries. Of the 10 clinical trials, 6 (60%) reported on at least one race. Of these six clinical trials, the enrollment rate of White, Asian, Black, and Hispanic ranged from 23.2%-78.4%, 13.6%-42%, 0.2%-5.2%, and 9.4%-12.1% respectively.

#### Racial subset enrollment because of disease prevalence among certain racial-ethnic population.

Among all 142 clinical trials, nine (6%) were influenced by disease prevalence among certain racial-ethnic population mostly because of epidermal growth factor receptor–activating mutations and Asian populations in lung cancer. Among these nine clinical trials, 5 (55.6%) reported on at least one race. Of these five clinical trials, the enrollment rate of White, Asian, Black, and Hispanic ranged from 23.2%-62.4%, 62.4%-76.5%, 0.2%-1.4%, and 1.9% – 2.5%, respectively.

#### Industry-sponsored versus cooperative group trials.

Among all 142 clinical trials, 136 (95.8%) were industry sponsored, 3 (2.1%) were cooperative group trials, whereas 3 (2.1%) were sponsored by other entities like National Cancer Institute, NCI. Of the 136 industry-sponsored trials, 71 (52.2%) reported on at least one race. Of these 71 clinical trials, the enrollment rate of White, Asian, Black, and Hispanic ranged from 31.9%-98.7%, 0.6%-65.4%, 0.18%-7.1%, and 0.93%-17.2%, respectively. Of the three cooperative group trials, 2 (66.7%) reported on at least one race, whereas of the three other sponsored trials, 1 (33.3%) reported on at least one race.

## DISCUSSION

During the 10-year time frame of our analysis (2009-2019), racial reporting in clinical trials leading to the approval of oral chemotherapy drug was identified in only about half of these clinical trials. The representation of BAs was consistently low relative to the cancer burden among this population. There was low representation of BAs remained consistently low regardless of enrollment locations and industry-sponsored versus cooperative group clinical trials. Asian people were more reasonably represented in clinical trials for tumors with EGFR activating mutations that can be more prevalent in locations with larger Asian populations. Regardless of enrollment locations, clinical trial sponsorship, or disease prevalence among certain racial-ethnic population, these medications are FDA approved in the United States for use of general population including BAs. This finding calls for more efforts to be made for reporting of racial representation in clinical trials as this will lead to better generalizability of study findings.

Several factors that affect BA participation in cancer-related clinical trials have been identified.^17^ For example, BAs are most likely to complain of mistrust of research and medical system as a barrier to participation in cancer-related clinical trials.^17^ Understanding these factors is critical for rectifying the disparities shown in our analysis. Lack of diversity in clinical trials decreases opportunities for identifying effects that could be particularly important to ethnic minority populations, thus leading to inappropriate distribution of benefits and risks of clinical trial participation.^17^ Increasing BA participation in clinical trials will lead to results that are generalizable to patients treated with the approved agents and higher quality of care for all patients.

In conclusion, BAs are under-represented in oral chemotherapy oncology clinical trials despite efforts made to increase minority participation in clinical trials. Considering the higher mortality rate found in this population in certain cancer types, there should be more BAs in cancer clinical trials to increase the generalizability of the results, improve outcomes, and eventually close the health disparity gap among this patient population.
